# Experimental Investigation of the Flow on a Simple Frigate Shape (SFS)

**DOI:** 10.1155/2014/818132

**Published:** 2014-01-12

**Authors:** Rafael Bardera Mora

**Affiliations:** INTA, Experimental Aerodynamics Branch, carretera de Ajalvir p. k. 4.5, 28850 Madrid, Spain

## Abstract

Helicopters operations on board ships require special procedures introducing additional limitations known as ship helicopter operational limitations (SHOLs) which are a priority for all navies. This paper presents the main results obtained from the experimental investigation of a simple frigate shape (SFS) which is a typical case of study in experimental and computational aerodynamics. The results obtained in this investigation are used to make an assessment of the flow predicted by the SFS geometry in comparison with experimental data obtained testing a ship model (reduced scale) in the wind tunnel and on board (full scale) measurements performed on a real frigate type ship geometry.

## 1. Introduction

Shipboard helicopter operations are performed in a very adverse and turbulent environment because the ship superstructure produces high airwake turbulence levels and the flight deck platform is short and is never static [1]. The interaction of the atmospheric wind and sea state with the ship creates the operational environment for the helicopter, different for every ship type [2], forcing the aircraft to land in nonideal conditions.

Operations on board ships require special procedures which introduce additional limitations know as ship helicopter operational limitations (SHOLs) [3, 4].

These limitations are not provided by the helicopter manufacturer, since they depend to a large extent on the ship involved and its environment. The development of tools to determine these limitations is a priority for navies [5, 6].

A typical case of study in experimental aerodynamics [7, 8] and computational simulations [9, 10] is the simple frigate shape (SFS). This case is a representative case of study of the flow on the ship flight deck, since it has a simplified geometry common to the main of frigates containing the most relevant aspects in an aerodynamic sense, as superstructure, bridge, hangar, and flight deck.

A SFS was manufactured and tested in a wind tunnel using experimental techniques as oil film visualization, PIV (particle image velocimetry), and LDA (laser doppler anemometry). Finally, the results provided testing the SFS geometry are assessed by comparing the results predicted by SFS with these obtained measuring on a frigate model tested in wind tunnel and measuring on board the real ship.

This investigation determines the capability of flow measurements over the SFS geometry in the prediction of airflow in the flight deck, which influences the ship environment on the helicopter capabilities, resulting in the flight envelope for maximum safe ship helicopter operational limitations (SHOL's) [3, 4].

## 2. Flight Deck Airflow

Flight deck is located downstream the hangar and dedicated to the helicopter operations as an helideck. One of the largest factors in the variation of flight deck wind flow is the ship structure that is, by definition, a bluff body which is defined as having a massive separated region in its wake at Reynolds number ranges of order 10^4^ and greater [11].

**Figure 1:**
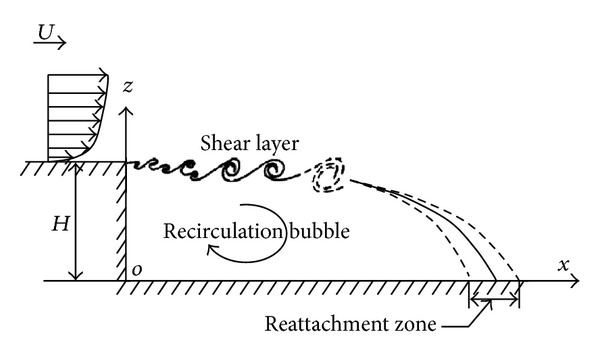
Two-dimensional flight deck flow field.

Two-dimensional flight deck flow field approximates to that of a backwards facing step (Figure 1), with a closed recirculation zone bounded by an unsteady shear layer emanating from the top of the hangar and reattaching on the flight deck [12].

**Figure 2:**
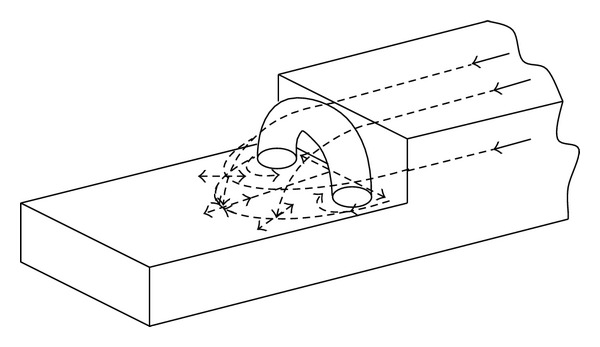
Three-dimensional flight deck flow field.

Extending the model into three dimensions requires to consider the flow perpendicular to the vertical face. the literature [13, 14] suggests a characteristic flow as shown in Figure 2 which has been observed through flow visualization tests. A large recirculation region behind the step is produced by the flow incoming to the flight deck from the sides of the ship and causing counterrotating vortices on each side of the recirculation region. The result is an unsteady horseshoe vortex structure [15]. This unsteadiness of the flow causes this structure to grow, dissipate, and move spatially in an unpredictable manner. Moreover, situations where the free stream has a crosswind component add a higher degree of complexity [16].

## 3. Simple Frigate Shape Model

Simple frigate shape (SFS) is a simplified geometry common to the main of frigates usually studied in a first step in both experimental aerodynamics [7, 8] and computational simulations [9, 10]. This geometry has the most relevant aspects in an aerodynamic sense, as superstructure, bridge, hangar, and flight deck.

**Figure 3:**
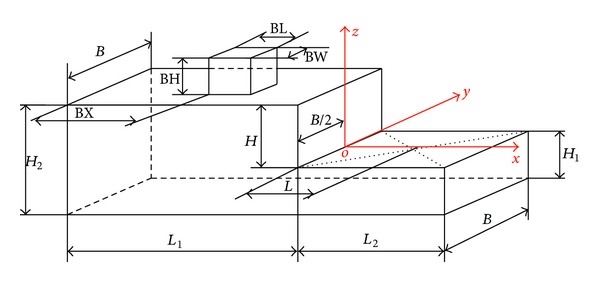
Simple frigate shape (SFS) dimensions.

Figure 3 shows a SFS geometry, where flight deck has a length *L*_2_ and is located downstream the hangar as a descendent step. Helicopter usually lands over the center of the flight deck, where the distance from the hangar is *L* (*L*_2_ = 2*L*) [12]. The hangar width (known as beam) is denoted by *B* and the hangar height (*H*) is usually used as a reference length. Usually the beam to hangar height ratio is from 2.0 to 2.5 and the length of the flight deck to hangar height ratio is around 3.0 or 4.0.

Finally, geometric dimensions were selected and a SFS was manufactured with a hangar height of 80 mm, a beam *B* = 2.5*H*, and a flight deck length *L*_2_ = 4.0*H*. The length precedent to the hangar *L*_1_ is 8.5*H*, height over the floor *H*_1_ is 0.75*H*, and the front height *H*_2_ is *H* + *H*_1_.

The bridge dimensions BL, BW, and BH were adapted from [9], and these values relative to the hangar height are the following: BX/*H* = 4.0, BL/*H* = 1.0, BW/*H* = 0.5, and BH/*H* = 1.0.

## 4. Wind Tunnel Setup

The experiments were conducted in a low-speed wind tunnel at INTA (Spain). The wind tunnel used is a closed-circuit wind tunnel type with an open test section of 2 × 3 m^2^ and a maximum airspeed of 60 m/s.

The wind tunnel has a platform with streamlined leading and trailing edges to minimize the interference of the platform in the flow field. In this test case the platform simulates the surface of the sea and hides the part of the hull underwater, without aerodynamic interference.

**Figure 4:**
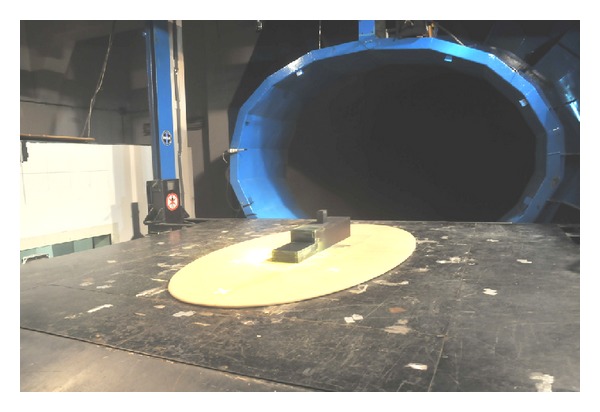
SFS model in the test section of the wind tunnel.

Figure 4 shows the SFS model in the test section of the wind tunnel and on the platform that simulates the surface of the sea.

## 5. Oil Film Visualization

The flow visualization over the surface of the flight deck was performed by means of the typical oil film technique [17]. The surface was coated with a thin layer of a specially prepared paint consisting of a mixture of alcohol, distilled water, and fluorescent powder pigment. When the wind tunnel blows due to the frictional forces, the air stream carries the liquid components of the paint and the remaining streaky deposit of the pigment gives information on the direction of the flow.

**Figure 5:**
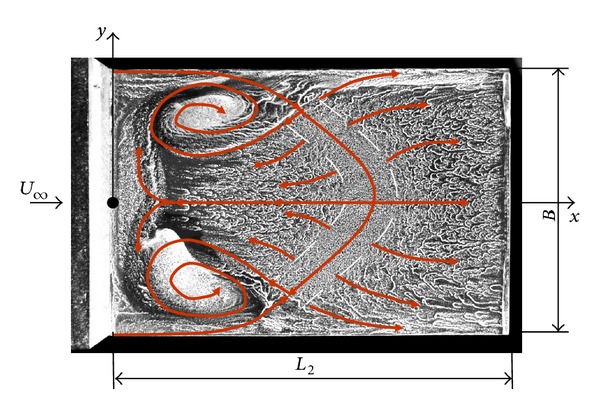
Flow pattern on the SFS flight deck surface.

In these tests, the wind was flowing into the tunnel during five minutes and we waited for several minutes until the paint was dried. The flow pattern was visualized and recorded by means of a photographic camera. The surface was illuminated by two black light tubes mod. Philips TLD 36W/08 in order to enhance the visualization pattern. The process was repeated three times to verify the repeatability of the flow pattern.  Figure 5 shows the results obtained when the airflow velocity was 20 m/s corresponding to a Reynolds number based on the hangar height of 1.1 · 10^5^ and zero angle of wind incidence. The flow pattern impressed on the surface shows different textures corresponding to different zones of the flow. The feet of the horseshoe vortex structure are visualized downstream the hangar. The intersection of the recirculation bubble with the flight deck is identified on a reattachment curved strip (dotted lines), and finally the flow motion over the flight deck surface is visualized.

## 6. Particle Image Velocimetry

The SFS flight deck flow was investigated by means of particle image velocimetry (PIV), that is a quantitative flow visualization technique used to obtain instantaneous flow field velocity measurements [18].

The freestream velocity *U*_*∞*_ was 20 m/s, corresponding to a Reynolds number based on the hangar height of 1.1 · 10^5^. The flow was seeded with olive oil tracer particles (1 *μ*m in diameter) [19] produced by an aeroso lgenerator based on Laskin nozzles [20]. The flow was illuminated by two Nd: YAG (neodymium: yttrium aluminium garnet) lasers with a maximum energy output of 190 mJoule per pulse and a pulse time separation of 15 *μ*s. The pulse width was 9 nanoseconds, so the flow motions could be frozen in a clear particle image.

A CCD (charge coupled device) camera, with a resolution of 2048^2^ pixels, in addition to an AF Micro-Nikkor 105 mm camera lens was used.

Spatial cross-correlation analysis computed via a 2D fast Fourier transform (FFT) [21] determines the average motion of the particles contained within regions known as interrogation windows.

PIV images processing was performed using an interrogation window of 32^2^ pixels with 50% window overlapped following the Nyquist sampling criteria. Correlation peak was located with subpixel accuracy by fitting a Gaussian curve [22].

Postprocessing analysis filling vector holes (spurious vectors) by a local mean filter size of 3 × 3 was applied.

PIV results are shown by typical maps [23] averaged over 50 instantaneous maps. Each map is obtained from a pair of flow images. After postprocessing, they were ensembleaveraged to get the spatial mean flow velocity maps. Streamlines are overlapped over the velocity maps (*H* is the hangar height).

**Figure 6:**
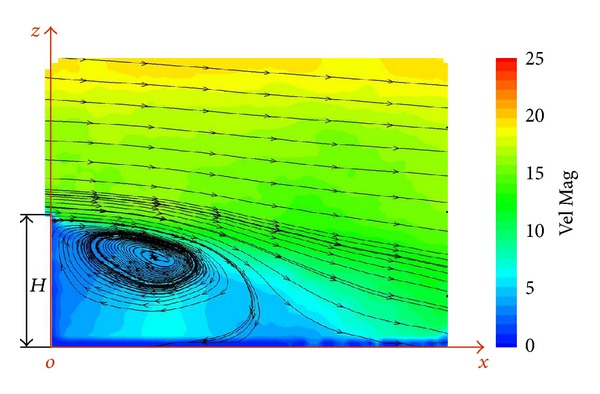
Velocity map in the vertical plane of symmetry of the SFS flight deck.

PIV measurements were performed with wind tunnel flow aligned to the longitudinal SFS axis (zero angle of wind incidence) and illuminating the vertical plane of symmetry of the deck (*y* = 0). The vortex structure was cut by this plane as are indicated by the streamlines in Figure 6.

A horizontal plane of the flow located at one-half of height (*z*/*H* = 0.5) of the SFS flight deck was investigated by means of PIV.

**Figure 7:**
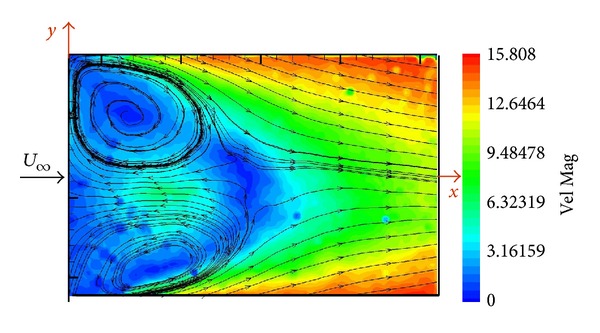
Velocity map in the horizontal plane of the SFS flight deck (*z*/*H* = 0.5).

Figure 7 shows the velocity map when the wind tunnel flow was aligned to the longitudinal ship axis.

Two eyes of the “*U*” vortex are visualized downstream the hangar. A recirculation bubble with very low velocity is located downstream the hangar behind the “*U*” vortex eyes. And finally at the end of the deck, velocity in the lateral edges has values of 75% of the free stream velocity but in the centre is only around the 50% of the free stream velocity.

**Figure 8:**
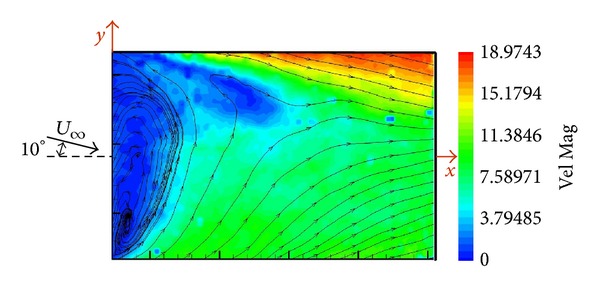
Velocity map in the horizontal plane of the SFS flight deck at 10° of angle of incidence (*z*/*H* = 0.5).

Similar to the previous case, Figure 8 shows the velocity map when the wind tunnel flow was coming with an angle of incidence of 10 degrees. The “*U* inverted” vortex structure downstream the hangar is now displaced and therefore only one eye of the vortex is visualized. The recirculation bubble is asymmetric and displaced. The velocity field has changed; the edge in the dark of the hangar has lower velocity values than the opposite edge.

**Figure 9:**
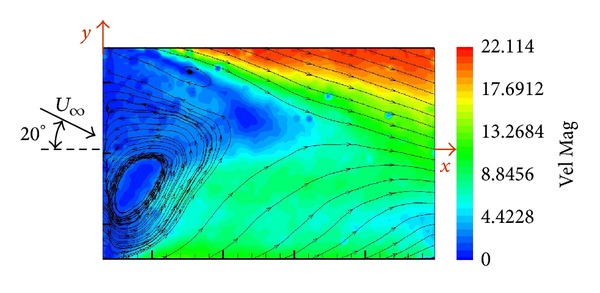
Velocity map in the horizontal plane of the SFS flight deck at 20° of angle of incidence (*z*/*H* = 0.5).

Now, Figure 9 shows the velocity map when the wind tunnel flow was coming with an angle of incidence of 20 degrees. The trend of the flow is the same as shown in Figure 8 but increased because the angle of incidence is higher. The displacement of the “*U* inverted” vortex structure downstream the hangar and the asymmetry of the recirculation bubble are visualized. Changes in the velocity field are stronger than the previous case; the edge in the dark of the hangar has lower velocity and the opposite edge has higher velocity.

## 7. Laser Doppler Anemometry

Laser Doppler anemometry (LDA) wind velocities measurements were carriedout in several points on a vertical line located on the centre of the flight deck.

Laser Doppler anemometry is a nonintrusive optical measurement technique used to measure the velocity at a point of the flow [24].

A commercial two-component laser Doppler anemometer from TSI, Inc., was used to measure simultaneously two independent velocity components.

Olive oil seeding particles 1 *μ*m in diameter [19] were injected into the settling chamber six meters upstream the wind tunnel test section.

Mean velocity in the centre of the SFS flight deck at several heights was measured as a function of the relative wind angle.

Two quantities are calculated from LDA measurements: nondimensional wind velocity and turbulence intensity.

Nondimensional wind velocity components are obtained from the LDA measurements after the following expressions:

(1) $$\begin{array}{c} \hat{u}=\frac{u}{V_{t}},\\ \hat{v}=\frac{v}{V_{t}}, \end{array}$$

where the symbol $\hat{\;}$ indicates nondimensional wind velocity components, *u* and *v* represent the mean value of velocity components measured by the LDA anemometer over the flight deck, and *V*_*t*_ is the modulus of the mean wind tunnel velocity blowing on the model.

Turbulence intensity was calculated from LDA measurements following [12], by dividing the standard deviation by the corresponding free stream velocity (not the local velocity magnitude) as follows:

(2) $$\begin{array}{c} I_{u}=\frac{\sigma_{u}}{V_{t}},\\ I_{v}=\frac{\sigma_{v}}{V_{t}}, \end{array}$$

where *I*_*i*_ and *σ*_*i*_ represent turbulence intensity and standard deviation of the “*i*” velocity component, respectively.

**Figure 10:**
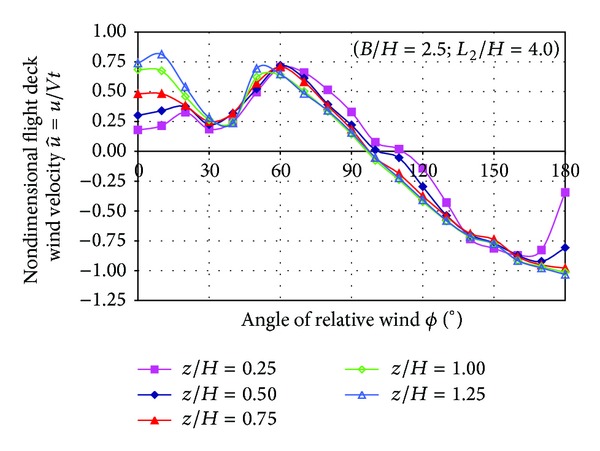
Nondimensional velocity $\hat{u}$ as a function of relative wind angle.

Figure 10 shows the plot of nondimensional $\hat{u}$ component of the velocity measured as a function of the relative wind angle for each nondimensional height (*z*/*H*). When the relative wind angle is higher of 30° all curves show the same trend indicating that $\hat{u}$ is approximately independent of the height (*z*/*H*). Higher velocity values (around 60% of the free stream velocity) in the positive branch of the curves are located in 60° while lower positive values are in a valley located in 30°.

**Figure 11:**
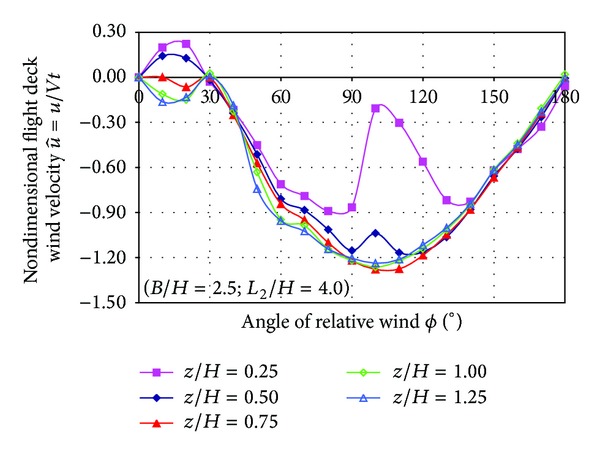
Nondimensional velocity $\hat{v}$ as a function of relative wind angle.

Figure 11 shows the plot of nondimensional $\hat{v}$ component of the velocity measured as a function of the relative wind angle for each nondimensional height (*z*/*H*). When the relative wind angle is higher of 30° all curves (except for *z*/*H* = 0.25) show the same trend indicating that $\hat{v}$ is approximately independent of the height (*z*/*H*).

The lowest values (around −130% of the free stream velocity) in the negative branch of the curve are located at 110°. Negative velocities are opposite to the axes shown in Figure 3.

**Figure 12:**
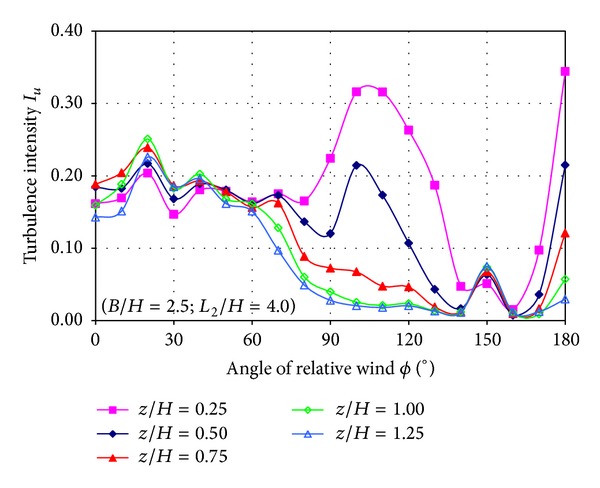
Turbulence intensity of the *u* velocity component as a function of relative wind angle.

Figure 12 shows the turbulence intensity of the *u* velocity component as a function of the relative wind angle for each nondimensional height (*z*/*H*). Several peaks of turbulence are found; the first peak is located at 20° for all heights measured, the second peak is very smooth and located at 40°, third peak is found at 110° but only in the heights of *z*/*H* = 0.25 and *z*/*H* = 0.50, and the fourth peak is found at 180° for all heights when the flow is coming from the stern (180°) of the ship.

Turbulence intensity values are around of 25% when the flow is coming with an incidence of 20° (first peak). When the angle is 40° (second peak) turbulence level is 20%. For a point located at *z*/*H* = 0.25 (close the floor) this increases above 30% when the angle of relative wind is 110°. When the flow is coming from the stern, the turbulence depends on the height, corresponding higher levels of turbulence to lower heights.

**Figure 13:**
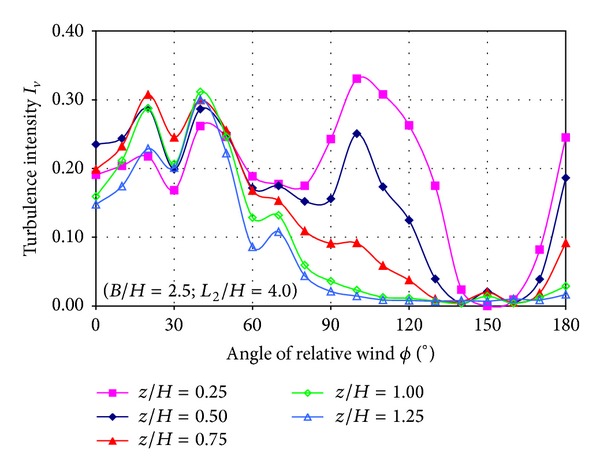
Turbulence intensity of the *v* velocity component as a function of relative wind angle.

Figure 13 shows the turbulence intensity of the *v* velocity component as a function of the relative wind angle for each nondimensional height (*z*/*H*). Several peaks of turbulence are found; the first peak is located at 20° and the second peak at 40° in all heights measured. The third peak is found at 100° but only for the heights of *z*/*H* = 0.25 and *z*/*H* = 0.50. And the fourth peak is found at 180° for all heights when the flow is coming from the stern of the ship.

Turbulence intensity levels are around 30% when the flow is coming from 20° and 40° (first and second peaks) but this increases up to 35% when the point is located at *z*/*H* = 0.25 (close the floor) for a relative wind angle of 100°. Finally, when the flow is coming from the stern, the turbulence depends on the height, corresponding higher levels of turbulence to lower heights.

The analysis of the two turbulence intensity components plots indicates that both turbulence levels are very similar and the locations of the turbulence peaks are approximately the same. Higher turbulence level is found for lower locations (near the floor).

Also, higher turbulence levels can be observed for 20°, 40°, and 100° of relative wind angle and when the flow is coming from the stern of the ship.

## 8. Measurements Comparison

In order to make a comparison analysis with a real frigate geometry, a reduced scaled 1 : 50th frigate model was fabricated with a length of 2000 mm and a beam of 313 mm. The hangar height defined as *H* is 125 mm, so the flight deck width is 2.5*H* (313 mm) and its length is 4.0*H* (500 mm).

**Figure 14:**
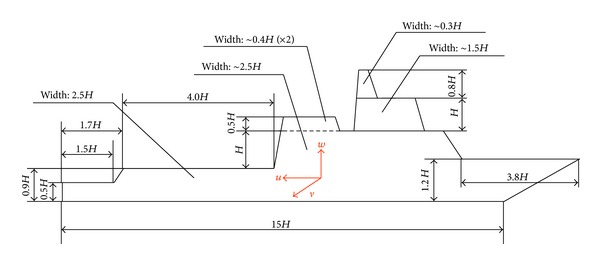
Sketch of the ship model.

Details of the ship model are given in Figure 14; all of them referred to hangar height *H* taken as reference length.

The flow on a real frigate type ship was investigated in both wind tunnel tests (model) and on board measurements (full scale) and the results were compared with those predicted by the simple frigate shape (SFS) wind tunnel tests.

**Figure 15:**
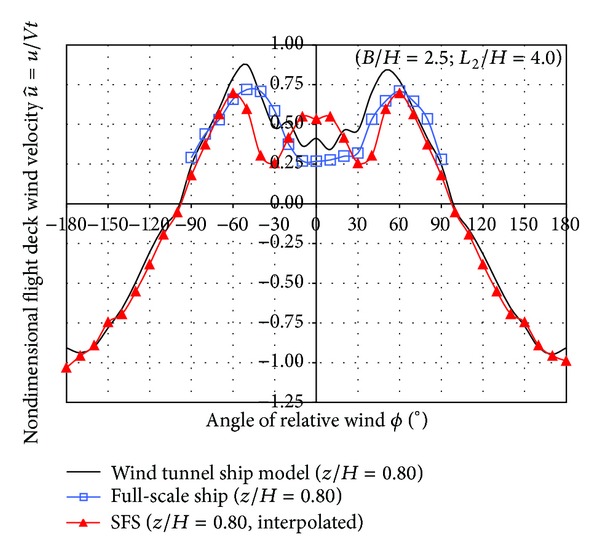
Comparison of the longitudinal nondimensional velocity.

**Figure 16:**
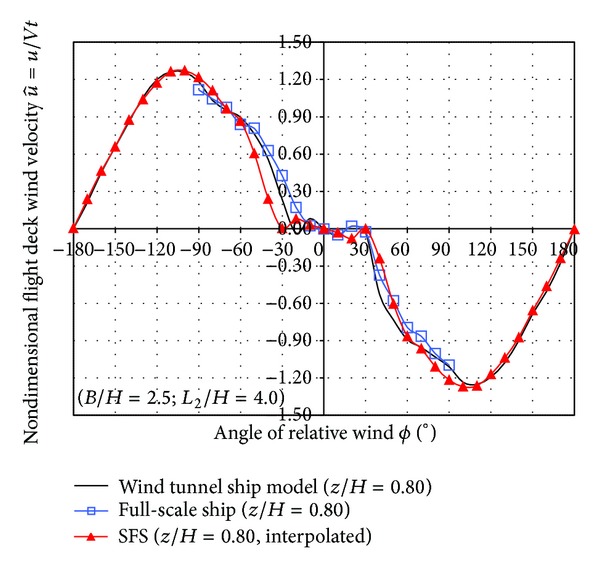
Comparison of the lateral nondimensional velocity.

Figures 15 and 16 show the comparison of the nondimensional velocities obtained in the three cases: SFS and ship model wind tunnel measurements and full scale on board measurements.

The SFS has a symmetric geometry, but the real frigates usually have slightly asymmetric geometry due to some devices as the stairs to descend from the hangar roof to the flight deck. This effect is observed in the wind tunnel ship model and on board measurements as shown in Figures 15 and 16.

Wind tunnel measurements on the frigate model were performed by laser Doppler anemometry in a point located at 0.10 meters height over the centre of the model flight deck. The wind tunnel velocity was 20 m/s corresponding to a Reynolds number based on the hangar height of 1.7 · 10^5^.

On board measurements were performed by a sonic three-component anemometer Metek mod. USA-1 located in the centre of flight deck over a mast 5 meters height. The resolution of this sonic anemometer in both wind velocity and wind direction is ±0.01 m/s and ±1°, respectively.

The ship measurements were done while the ship course was fixed during 15 minutes and the relative wind angle was incremented by steps of 10 degrees in the range from −90° to +90°, as usual. The mean ship velocity during the experiments was 4 m/s (~8 knots). The averaged relative wind velocity blowing on the ship at zero angle of wind incidence was 9.41 m/s.

As previously stated, Figure 15 shows the comparison of the longitudinal nondimensional velocity component at a point located at *z* = 0.80*H* of height on a vertical line located on the centre of the flight deck. Results from wind tunnel ship model show the same trend as full-scale ship results, showing approximately a good agreement.

Results obtained by SFS overestimate the $\hat{u}$ values in the range from −10° to +10°. Conversely, when the range is between 30° and 60° (equivalently in the negative branch) the trend is correct but the values are lower than on board and ship model measurements.

Similarly, Figure 16 shows the same comparison of the lateral nondimensional velocity component at a point located at *z* = 0.80*H* of height on a vertical line located on the centre of the flight deck.

Results from wind tunnel ship model show the same trend as full-scale ship results, showing approximately a good agreement.

Results obtained by SFS show similar trend with those obtained in wind tunnel ship model and on board full-scale ship, but in the negative branch slight differences are observed. In the range from −30° to −60° for the negative branch of SFS, lower values are showed compared with these obtained in both wind tunnel ship model and on board full-scale ship.

## 9. Conclusions

Helicopter operations on board ships require special procedures because they are performed in a very adverse and turbulent environment. The ship superstructure produces high airwake turbulence levels and the platform of the flight deck which is short and never static introduces additional limitations known as ship helicopter operational limitations (SHOLs). These limitations are not provided by the helicopter manufacturer, since they depend to a large extent on the ship involved and its environment. The development of tools to determine these limitations is a priority for all navies.

A typical and representative case of study of the flow on the ship flight deck in experimental aerodynamics and computational simulations is the simple frigate shape (SFS), since it contains the most relevant geometry aspects common to the main frigates in an aerodynamic sense.

A SFS was fabricated and tested in a wind tunnel using several experimental techniques, as oil film visualization, PIV (particle image velocimetry), and LDA (laser Doppler anemometry).

Oil film visualization shows the flow pattern structure on the flight deck SFS surface visualizing the vortex eyes and the reattachment of the recirculation bubble.

The results of the investigation by means of particle image velocimetry in both vertical and horizontal planes of the flight deck flow are shown by velocity maps. The horseshoe “*U*” inverted structure was identified and the flow field velocity measured.

Laser Doppler anemometry was used to investigate point by point the flow velocity and turbulence levels in a vertical line located in the centre of the flight deck. This line is considered as the most hazardous of the helicopters path because it is strongly influenced by the ship environment.

On board measurements (fullscale) were performed by a sonic three-component anemometer located in the centre of flight deck over a mast 5 meters height while the ship course was fixed.

Finally, the results provided by testing the SFS geometry are assessed by comparing the results predicted by this SFS geometry with these obtained measuring in wind tunnel on a frigate model and measuring on board the real ship at a full scale. The comparison analysis indicates that although SFS results predict correctly the ship frigate flow in a wide range of wind incidence angles the application of the SFS results to real frigates geometry must be performed taking into account that the SFS results differ slightly in a short range, located approximately around zero wind incidence and at ±30–60°.

Inconclusion, this investigation determines the capability of the simple frigate shape (SFS) geometry in the prediction of the airflow in the flight deck which influences the ship environment on the helicopter capabilities resulting in the flight envelope for maximum safe ship helicopter operational limitations (SHOL's).

## Nomenclature

*B*: Hangar width or beam
BH: Bridge height
BL: Bridge length
BW: Bridge width
BX: Bridge location
CCD: Charge coupled device
FFT: Fast Fourier transform
*H*: Height of the hangar
*I*_*i*_: Turbulence intensity of the “*i*” velocity component
*L*: Distance from the hangar to the centre of the flight deck
*L*_1_: Length of the SFS precedent to the hangar
*L*_2_: Length of the flight deck
Nd: YAG: Neodymium: yttrium aluminium garnet
PIV: Particle image velocimetry
SFS: Simple frigate shape
SHOL: Ship helicopter operational limitations
*U*: Mean value of the *x* velocity component measured by LDA
*U*_*∞*_: Freestream velocity
*v*: Mean value of the *y* velocity component measured by LDA
*V*_*t*_: Modulus of the mean wind tunnel velocity blowing on the model
*σ*_*i*_: Standard deviation of the “*i*” velocity component
$\hat{\;}$: Nondimensional wind velocity component.
